# Interplay between Inter-Subunit Rotation of the Ribosome and Binding of Translational GTPases

**DOI:** 10.3390/ijms24086878

**Published:** 2023-04-07

**Authors:** Ananya Das, Nichole Adiletta, Dmitri N. Ermolenko

**Affiliations:** Department of Biochemistry & Biophysics, School of Medicine and Dentistry & Center for RNA Biology, University of Rochester, Rochester, NY 14642, USA

**Keywords:** ribosome, inter-subunit rotation, EF-G, single-molecule Förster Resonance Energy Transfer (smFRET)

## Abstract

Translational G proteins, whose release from the ribosome is triggered by GTP hydrolysis, regulate protein synthesis. Concomitantly with binding and dissociation of protein factors, translation is accompanied by forward and reverse rotation between ribosomal subunits. Using single-molecule measurements, we explore the ways in which the binding of translational GTPases affects inter-subunit rotation of the ribosome. We demonstrate that the highly conserved translation factor LepA, whose function remains debated, shifts the equilibrium toward the non-rotated conformation of the ribosome. By contrast, the catalyst of ribosome translocation, elongation factor G (EF-G), favors the rotated conformation of the ribosome. Nevertheless, the presence of P-site peptidyl-tRNA and antibiotics, which stabilize the non-rotated conformation of the ribosome, only moderately reduces EF-G binding. These results support the model suggesting that EF-G interacts with both the non-rotated and rotated conformations of the ribosome during mRNA translocation. Our results provide new insights into the molecular mechanisms of LepA and EF-G action and underscore the role of ribosome structural dynamics in translation.

## 1. Introduction

Protein synthesis is accompanied by ~10° forward and reverse rotation between the small (30S in bacteria) and large (50S in bacteria) ribosomal subunits [1]. The inter-subunit rotation was discovered in cryo-EM reconstructions of the ribosome bound with the catalyst of mRNA-tRNA translocation, universally conserved elongation factor G (EF-G) [2]. The following structural and single-molecule fluorescence studies showed that spontaneous movement of peptidyl- and deacylated tRNAs into the hybrid A/P and P/E sites, which occurs during each elongation cycle after the formation of new peptide bond [3], is coupled to the transition from the non-rotated (NR) to rotated (R) conformation of the ribosome [4,5]. EF-G transiently stabilizes the R, hybrid state conformation, and then induces mRNA-tRNA translocation on the small ribosomal subunit that is coupled to reverse rotation between ribosomal subunits [4,5].

Acylation state of P-site tRNA plays a critical role in control of inter-subunit rotation. The presence of peptidyl-tRNA in the P site fixes the ribosomes in the NR conformation [6]. Deacylation of P-site tRNA by peptidyl transfer during the elongation phase of protein synthesis or peptide release during translation termination enable spontaneous inter-subunit rotation [6,7,8].

The binding of translation and inter-subunit dynamics is linked in the intricate interplay that is yet to be fully understood. On the one hand, translation factors perturb the equilibrium between different states of inter-subunit rotation. For example, initiation factor 2 (IF2), release factors 1 and 2 (RF1 and RF2) and ribosome recycling factor (RRF) were demonstrated to stabilize the semi-rotated, non-rotated and rotated conformations of the ribosome, respectively [8,9,10,11,12]. On the other hand, the acylation state of P-site tRNA, which controls inter-subunit dynamics, might regulate binding of translation factors to the ribosome. In particular, the binding sites of translational G proteins (EF-Tu, EF-G, RF3, IF2, LepA and BipA) extensively overlap. GTPase activity of these factors is invariably triggered by interaction with the sarcin–ricin loop (SRL) of 23S rRNA of the 50S subunit [1]. At least in part, the correct order for the binding of translational GTPases might be regulated by conformational rearrangements of the ribosome, including oscillation between the NR and R conformations.

In this work, we examine the ways in which the binding of ribosome GTPases LepA and EF-G affects the equilibrium between the NR and R conformations of the ribosome. We also explore the regulation of EF-G binding by acylation state of P-site tRNA. Our results provide new insights into relationship between inter-subunit rotation and binding of translational GTPases.

## 2. Results

### 2.1. LepA Stablizes the Non-Rotated Conformation of the Ribosome

LepA is a ribosome-dependent GTPase that is highly conserved among bacteria, eukaryotic mitochondria and chloroplasts. Paradoxically, despite the high levels of conservation, deletion of genes encoding LepA in *Escherichia coli* [13,14] and its yeast homologue Guf1 in *Saccharomyces cerevisiae* [15] have no phenotype under normal growth conditions. Knockout of mitochondrial homologue of LepA in mice causes male infertility [16].

Initially, bacterial LepA was described as a back-translocase that induces reverse translocation of tRNA and mRNA in the ribosome [17]. Subsequent studies implicated LepA in 30S subunit biogenesis [18]. In addition, several studies indicated that LepA is associated with the cell membrane [15,19] and is needed for synthesis of a number of membrane-associated proteins [20]. Thus, the role of LepA in translation remains elusive.

Cryo-EM and X-ray crystal structures of LepA-ribosome complexes revealed extensive interactions of C-terminal domain of LepA with acceptor stems of both P-site and A-site tRNAs [21,22,23]. Furthermore, structures of LepA-ribosome complexes revealed 4–5° clockwise rotation of the small ribosomal subunit relative to the large subunit when the ribosome was viewed from the solvent side of the 30S subunit and compared to the non-rotated, classical state conformation [21,22]. This rearrangement is distinct from the counterclockwise rotation observed in the rotated, hybrid state conformation of the ribosome.

To gain new insights into LepA function and to test whether LepA induces a distinct conformation of the ribosome, we employed single-molecule Förster Resonance Energy Transfer (smFRET) microscopy. Using smFRET, we examined the effects of LepA binding on ribosome inter-subunit dynamics. Inter-subunit rotation was followed by energy transfer between acceptor (Cy5) fluorophore attached to protein bS6 on the small subunit and donor (Cy3) attached to protein bL9 on the large subunit [6,11,24]. Numerous independent studies have previously shown that the NR and R conformations of the ribosome correspond to 0.6 and 0.4 FRET states of this FRET pair [6,11,24].

**Figure 1:**
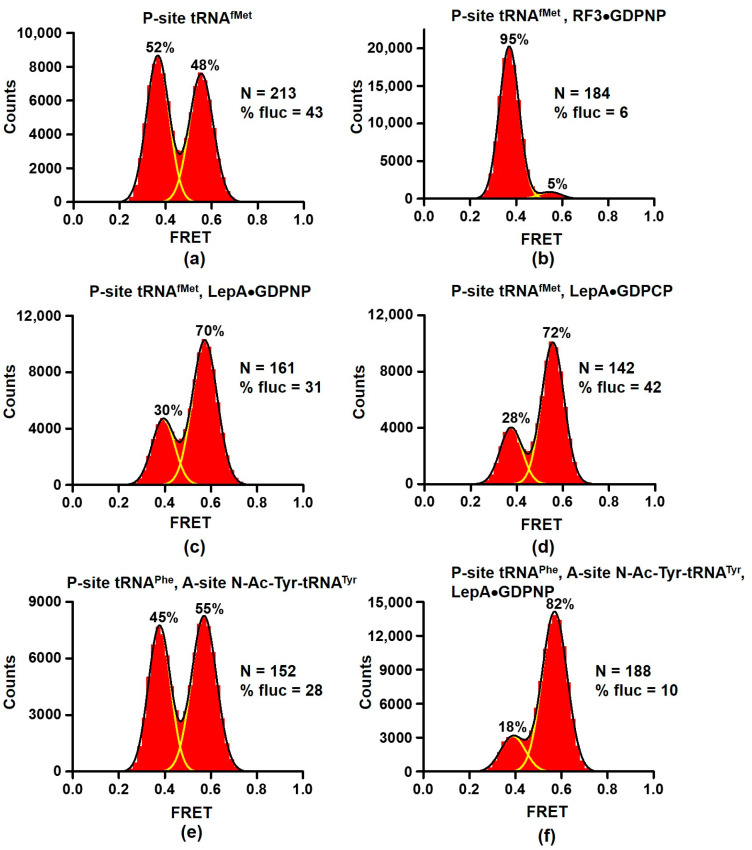
LepA stabilizes the non-rotated conformation of the ribosome. Histograms show FRET distribution in S6-Cy5/L9-Cy3 ribosomes. Ribosomes bound with P-site deacylated tRNA^fMet^ (**a**–**d**) were incubated with RF3•GDPNP (**b**) or LepA•GDPNP **(c)** or LepA•GDPCP (**d**). Ribosomes bound with P-site deacylated tRNA^Phe^ and A-site N-Ac-Tyr-tRNA^Tyr^ (**e**,**f**) were incubated with LepA•GDPNP (**f**). N indicates the total number of FRET traces compiled into each histogram. % fluc designates the percentage of traces that show fluctuations. Yellow lines show individual Gaussian fits. Black line indicates the sum of Gaussian fits. The fraction of the ribosomes in the R and NR conformations are shown above the corresponding 0.37 and 0.56 Gaussian peaks.

We non-enzymatically bound deacylated tRNA^fMet^ to the P site of S6-Cy5/L9-Cy3 ribosomes programmed with a short-model mRNA. Previous studies demonstrated that ribosomes containing deacylated tRNA^fMet^ in the P site are more dynamic than complexes assembled with elongator tRNAs [6,25]. tRNA^fMet^-bound ribosomes exhibit multiple spontaneous fluctuations between the NR and R conformations of the ribosome in the absence of factors of translation [6]. In agreement with published data, the NR (0.6 FRET) and R (0.4 FRET) conformations were nearly equally populated in ribosomes containing P-site tRNA^fMet^ (Figure 1a). Over 40% of ribosomes showed spontaneous fluctuations between 0.4 and 0.6 FRET states.

To test sensitivity of our experimental setup to perturbation of NR↔R equilibrium, we incubated S6-Cy5/L9-Cy3 ribosomes containing P-site tRNA^fMet^ with 2 μM release factor 3 (RF3) and non-hydrolysable analogue of GTP, GDPNP, which traps RF3 on the ribosome. RF3 stimulates dissociation of release factors 1 and 2 (RF1 and RF2) from post-termination ribosome and stabilizes the R conformation of the ribosome [8,12,26]. Consistent with previous reports, nearly all (95%) of RF3-bound ribosomes were observed in the R conformation (Figure 1b). Furthermore, RF3 binding dramatically diminished the number of traces showing spontaneous fluctuations (Figure 1b).

When S6-Cy5/L9-Cy3 ribosomes containing P-site tRNA^fMet^ were incubated with 2 μM LepA and one of non-hydrolysable analogues of GTP, GDPNP or GDPCP, the NR↔R equilibrium shifted toward the NR conformation as 70% of ribosomes were observed in 0.6 FRET state. Importantly, we did not detect an additional high FRET state that would be consistent with clockwise inter-subunit rotation observed in X-ray crystal structures of LepA-ribosome complexes.

Because LepA was shown to interact with P-site and A-site tRNAs, we next assembled pre-translocation ribosomes that contained deacylated tRNA^Phe^ in the P site and peptidyl-tRNA analogue N-acetyl(Ac)-Tyr-tRNA^Tyr^ in the A site (Figure 1e). Incubation with LepA and GDPNP led to significant stabilization of the NR conformation as the fraction of ribosomes showing 0.6 FRET increased from 55% to 82% (Figure 1e,f).

Taken together, our data do not support the idea that LepA induces a unique conformation of the ribosome. Instead, LepA stabilizes the NR, classical-state conformation of the ribosome.

### 2.2. EF-G interacts with Both the Rotated and Non-Rotated Conformations of the Ribosome

Universally conserved translational GTPase EF-G (EF-2 in eukaryotes) catalyzes codon-by-codon ribosome translocation along mRNA. Early cryo-EM structures revealed EF-G bound to the R, hybrid-state conformation of the ribosome [2,27]. In addition, biochemical experiments suggested that the presence of P-site peptidyl-tRNA, which fixes the ribosome in the NR conformation, essentially prevents EF-G binding [28]. These observations together with several smFRET studies [29,30] led to the model that EF-G binding is restricted to the R, hybrid-state conformation of the ribosome. Other single-molecule and ensemble kinetic studies argued that EF-G can bind to NR ribosomes, transiently induce the R conformation before completion of translocation coupled to the return to the NR conformation [31,32,33,34] (detailed discussion of these models can be found in [35]).

**Figure 2:**
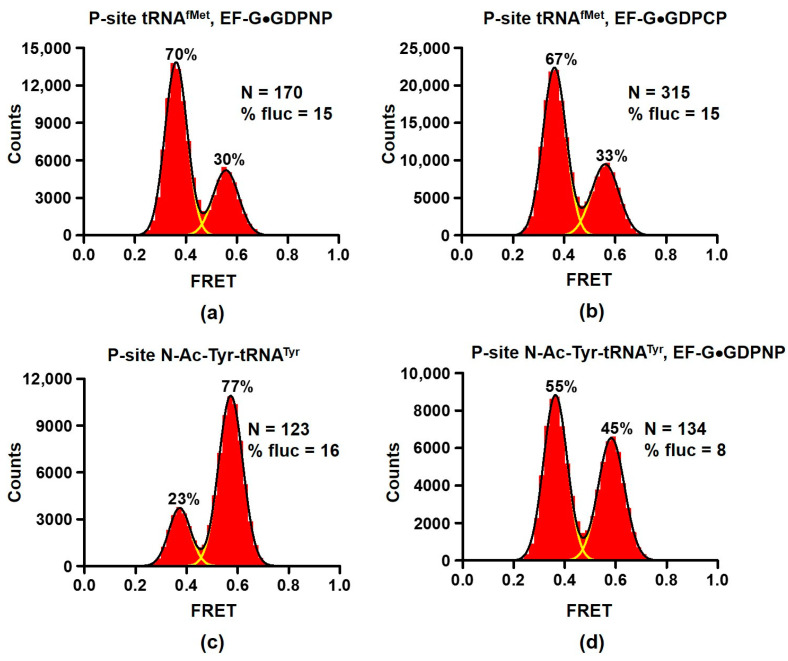
EF-G stabilizes the rotated conformation of the ribosome. Histograms show FRET distribution in S6-Cy5/L9-Cy3 ribosomes. Ribosomes bound with P-site deacylated tRNA^fMet^ (**a**,**b**) were incubated with either EF-G•GDPNP (**a**) or EF-G•GDPCP (**b**). Ribosomes bound with P-site acylated N-Ac-Tyr-tRNA^Tyr^ (**c**,**d**) were incubated with EF-G•GDPNP (**d**). N indicates the total number of FRET traces compiled into each histogram. % fluc designates the percentage of traces that show fluctuations. Yellow lines show individual Gaussian fits. Black line indicates the sum of Gaussian fits. The fraction of the ribosomes in the R and NR conformations are shown above the corresponding 0.37 and 0.56 Gaussian peaks.

We re-examined the effect of EF-G binding on the NR↔R equilibrium. When EF-G was incubated with ribosomes containing deacylated tRNA^fMet^ in the P site (Figure 1a) in the presence of either GDPNP or GDPCP, ~70% of ribosomes exhibited 0.4 FRET consistent with stabilization of the R conformation by EF-G (Figure 2a,b). Importantly, mRNA translocation requires the presence of both A- and P-site tRNAs [36]. Therefore, our smFRET measurements in ribosomes bound with a single deacylated P-site tRNAs reflect changes in the NR↔R equilibrium of pre-translocation ribosomes rather than switching from pre- to post-translocation conformation state of the ribosome.

Noteworthy, EF-G was present at concentration (2 μM) that is two orders of magnitude higher than reported K_D_ for EF-G binding [37]. Nevertheless, 30% of ribosomes were observed in the NR conformation and 15% of ribosomes showed fluctuations between 0.4 and 0.6 FRET states. For comparison, RF3 binding produced much stronger stabilization of the R conformation as only ~5% of ribosomes showed 0.6 FRET and spontaneous FRET fluctuations (Figure 1b). These results are consistent with smFRET experiments demonstrating prevalence of tRNA fluctuations between classical and hybrid states of tRNA binding coupled to the L1 stalk movement that were observed in ribosomes bound with EF-G•GDPNP [25].

Next, we tested whether EF-G interacts with ribosomes containing peptidyl-tRNA in the P site. When EF-G•GDPNP was incubated with ribosomes containing peptidyl-tRNA analogue N-Ac-Tyr-tRNA^Tyr^ in the P site, the fraction of ribosomes in the NR (0.6 FRET) conformation decreased from 77 to 45% (Figure 2c,d). This result indicates that EF-G interacts ribosomes containing peptidyl-tRNA in the P site and switches some of them from the NR to R conformation.

**Figure 3:**
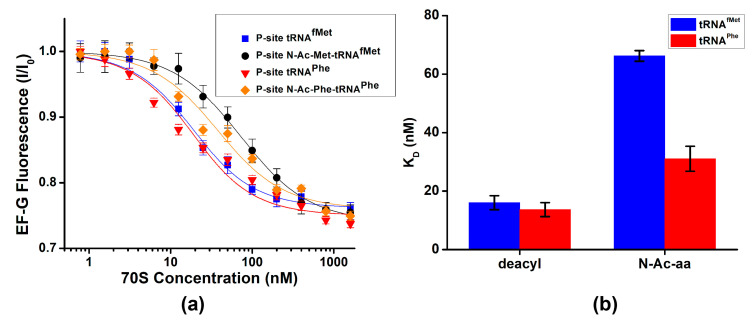
Deacylation of P-site tRNA moderately enhances EF-G binding. (**a**) EF-G fluorescence (I) normalized to fluorescence observed in the absence of the ribosome (I_0_) was measured with increasing concentrations of ribosome complex P-site tRNA^fMet^ (blue), N-Ac-Met-tRNA^fMet^ (black), tRNA^Phe^ (red) or N-Ac-Phe-tRNA^Phe^ (orange). (**b**) Dissociation constants (K_D_) of EF-G binding determined using fluorescein quenching by ribosomes that contain in the P site either deacyl- or N-Ac-aminoacyl-tRNAs.

To further investigate the ways in which the presence of peptidyl-tRNA in the P site affects EF-G binding, we measured dissociation constants of EF-G binding using previously established equilibrium titration assay [37]. Fluorescein-labeled EF-G was incubated with increasing amounts of 70S ribosomes containing either deacylated or peptidyl-tRNA in the P site. Fluorescein quenching indicates ribosome binding (Figure 3). Dissociation constants for EF-G binding to ribosomes containing deacylated tRNA^fMet^ or tRNA^Phe^ were 16 and 14 nM, respectively (Figure 3a,b, Table 1). The presence of N-acetyl (Ac)-aminocyl moiety at the acceptor stem of P-site tRNA increased the dissociation constant of EF-G binding by 2 (N-Ac-Phe-tRNA^Phe^)–4 (N-Ac-Met-tRNA^fMet^)-fold (Figure 3b, Table 1). Therefore, our data demonstrate that P-site peptidyl-tRNA only moderately weakens EF-G binding relative to deacylated tRNA.

**Figure 4:**
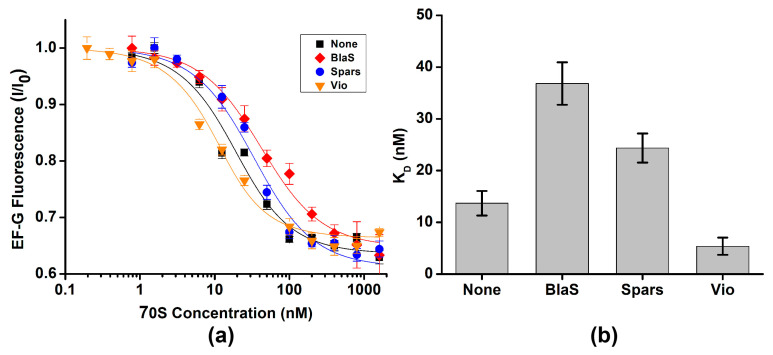
Antibiotics known to perturb the NR↔R equilibrium mildly affect EF-G binding. (**a**) EF-G fluorescence (I) normalized to fluorescence observed in the absence of the ribosome (I_0_) was measured with increasing concentrations of ribosome complexes containing P-site tRNA^Phe^ in the presence of 200 μM blasticidin S (BlaS, red), 40 μM sparsomycin (Spars, blue), 500 μM viomycin (Vio, orange) or in the absence of antibiotics (none, black). (**b**) Dissociation constants (K_D_) of EF-G binding determined in the presence or absence of antibiotics.

Next, we explored whether antibiotics that are known to stabilize either the NR or R conformation affect EF-G binding. Antibiotics blasticidin S and sparsomycin interact with the acceptor end of P-site tRNA and stabilize the ribosomes in the NR conformation by enhancing binding of CCA end of deacylated tRNA to the 50S P site and inhibiting tRNA translocation into P/E hybrid state [38,39]. Pre-incubation of ribosomes containing deacylated tRNA^Phe^ in the P site with either blasticidin S or sparsomycin increased the dissociation constant of EF-G binding by ~2-fold (Figure 4a,b, Table 1). These results were similar to moderate reduction in EF-G affinity toward the ribosome containing peptidyl-tRNA in the P site (Figure 3).

In contrast to blasticidin S and sparcomycin, translocation inhibitors viomycin traps the ribosome in the R conformation [6,40]. Pre-incubation of ribosomes containing deacylated tRNA^Phe^ in the P site with viomycin decreased the apparent dissociation constant for EF-G binding to 5 nM. Because EF-G concentration in titration experiments was 10 nM, 5 nM value of the apparent dissociation constant indicates stochiometric mode of EF-G binding. We could not perform titration experiments at lower EF-G concentrations to accurately determine dissociation constant for EF-G binding in the presence viomycin because of experimental limitation, i.e., fluorimeter sensitivity and necessity to use small sample volumes. Hence, 5 nM value represents an upper boundary for dissociation constant of EF-G binding in the presence of viomycin. Nevertheless, our results indicate that viomycin enhances EF-G binding to the ribosome that is consistent with early non-equilibrium measurements [41].

## 3. Discussion

**Figure 5:**
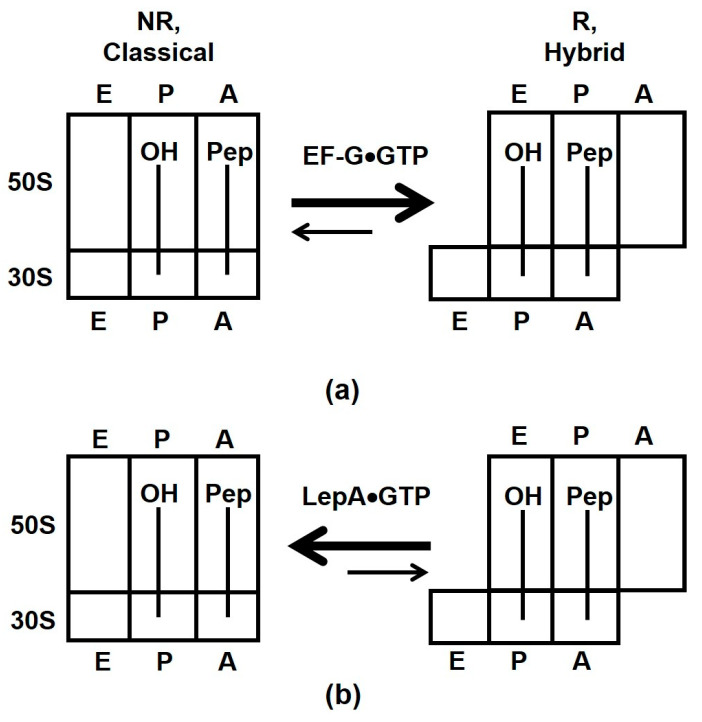
Effects of EF-G (**a**) and LepA (**b**) binding on the equilibrium between the NR, classical and R, hybrid states of the ribosome. Letters E, P and A indicate exit, peptidyl and aminoacyl tRNA binding sites on the 30S and 50S subunits.

Our equilibrium titration experiments show that stabilization of the NR conformation of the ribosome by the presence of polypeptide moiety of P-site tRNA or antibiotics sparsomycin and blasticidin S leads to 2–4-fold reduction in ribosome affinity for EF-G. Stabilization of the R, hybrid-state conformation by viomycin enhances EF-G binding. Nevertheless, EF-G is present in *E. coli* cells at 50–100 μM concentration [42], which is about three orders of magnitude higher than dissociation constants of EF-G binding measured in our equilibrium titration experiments (Table 1). Hence, switching between the R and NR conformations of the ribosome unlikely results in “on–off” regulation of EF-G binding. Hence, our results support the model suggesting that EF-G binds both the R and NR conformations of the pre-translocation ribosome [35]. Upon binding to NR ribosomes, EF-G induces the R, hybrid-state conformation [32] that is consistent with stabilization of the R conformation of the ribosome in equilibrium smFRET experiments (Figure 2 and Figure 5a).

The ability of EF-G to interact with both NR and R conformations is also important for inducing translocation of mRNA and tRNAs on the 30S subunit. mRNA-tRNA translocation is coupled to the reverse inter-subunit rotation from the R to NR conformation upon which domain IV of EF-G displaces peptidyl-tRNA in the A-site [1,2,32,43,44,45]. The reverse inter-subunit rotation also coincides with dissociation of the GTPase domain from the SRL followed by EF-G release from the ribosome [1,46]. However, non-hydrolysable analogues of GTP (i.e., lack of GTP hydrolysis) decouple the reverse inter-subunit rotation from EF-G release [1,44].

Our smFRET experiments with another ribosome GTPase, LepA, showed that this factor stabilizes the NR conformation of the ribosome (Figure 1c–f and Figure 5b). Our results provide no supporting evidence for 4–5° clockwise rotation of the small ribosomal subunit relative to the large subunit observed in X-ray crystal and cryo-EM structures of LepA-ribosome complexes [21,22]. However, our results are consistent with the cryo-EM reconstruction of LepA bound to NR ribosomes [47].

The NR conformation was most prevalent in LepA-bound ribosomes in the presence of both A- and P-site tRNAs (Figure 1). This observation is consistent with cryo-EM and X-ray crystal structures of LepA-ribosomes complexes that demonstrate interactions of C-terminal domain of LepA with both A- and P-site tRNAs [21,22,23]. These structures together with our smFRET data suggest that LepA interacts with the ribosome during elongation phase of protein synthesis. Since LepA was implicated in synthesis of membrane-associated proteins [20], we speculate that LepA is involved in membrane-tethered protein synthesis mediated by the insertase of Oxa1/Alb3/YidC protein family, which directs the biogenesis of membrane proteins in mitochondria, chloroplasts and bacteria, respectively [48]. Structural data indicate that LepA distorts the acceptor step of A-site tRNA away from the peptidyl-transferase center of the 50S subunit [21,22,23]. Perhaps, LepA-induced distortion of A-site peptidyl-tRNA pulls nascent peptide inside the ribosome exit tunnel to promote polypeptide backtracking or to mechanically unfold nascent polypeptide that might facilitate membrane tethering [49].

## 4. Materials and Methods

**Buffer systems.** Experiments were performed in polyamine buffer: 50 mM HEPES, pH 7.5, 6 mM MgCl_2_, 150 mM NH_4_Cl, 6 mM β-mercaptoethanol, 0.1 mM spermine and 2 mM spermidine.

**Preparation of ribosome, translation factors, tRNA and mRNA**. Cy3 and Cy5 maleimide were purchased from Click Chemistry Tools; GDPNP—Jena Biosciences (Jena, Germany); GDPCP—Sigma Aldrich (St. Louis, MI, USA). S6-Cy5/L9-Cy3 ribosomes were prepared by partial reconstitution of ΔS6-30S and ΔL9-50S *E. coli* subunits with S6-41Cys-Cy5 and L11-11Cys-Cy3 proteins as previously described [11,50]. tRNA^fMet^, tRNA^Phe^ and tRNA^Tyr^ were purchased from Chemical Block (Moscow, Russia). tRNAs were aminoacylated using S100 cell extract; Tyr-tRNA^Tyr^ and Met-tRNA^fMet^ were acetylated as previously described [40]. EF-G with His_6_ C-terminal tag was purified using affinity chromatography followed by Superdex-200 gel filtration [51]. LepA with a TEV protease-cleavable His_6_ N-terminal tag was expressed and purified as previously described [52]. The plasmid encoding histidine-tagged LepA was a gift from Clint Spiegel.

Model mRNAs HIV_NSΔFSS [53] and m291 [36] were transcribed using run-off transcription catalyzed by T7 RNA polymerase according to standard procedures. HIV_NSΔFSSmRNA: 5′ *GGUUUUUCUUCUGAAGAUAAAG*CAACAACAACAAGGC**AAGGAGG**AAAAAUGUUCUACAA 3′ (AL2-complementray sequence is italicized; Shine–Dalgarno sequence is in bold; AUG codon is underlined). mRNA m291: 5′ GUAAAGUGUCAUAGCACCAACUGUUAAUUAAAUUAAAUUAAA**AAGGA**AAUAAAAAUGUUUGUAUACAAAUCUACUGCUGAACUCGCUGCACAAAUGGCUAAACUGAAUGGCAAUAAA*GGUUUUUCUUCUGAAGAUAAAG* 3′ (AL2-complementray sequence is italicized; Shine–Dalgarno sequence is in bold; AUG codon is underlined).

**Ribosome complex assembly for smFRET experiments.** To fill P site of the ribosome, 0.3 μM S6/L9-labeled 70S ribosomes were incubated with either 0.9 μM tRNA^fMet^, tRNA^Phe^ or N-Ac-Tyr-tRNA^Tyr^ in the presence of 0.6 μM mRNA for at 37 °C 10 min. HIV_NS ΔFSS mRNA was used to assemble ribosome complexes with P-site tRNA^Phe^ or N-Ac-Tyr-tRNA^Tyr^; m291 mRNA was used to assemble complexes with P-site tRNA^fMet^. HIV_ΔFSS and 291 mRNAs were pre-annealed to 3′ and 5′ biotin-AL2 DNA oligo, respectively (AL2 oligo: 5′ CTTTATCTTCAGAAGAAAAACC 3′). To bind aminoacyl-tRNA to the A site, 0.6 μM N-Ac-Tyr-tRNA^Tyr^ was incubated with ribosomes containing P-site tRNA^Phe^ at 37ºC for 20 min. Ribosomal complexes were diluted to 2 nM before smFRET imaging.

**smFRET measurements.** Quartz slides were coated with dichlorodimethylsilane (DDS), bound with biotin-BSA and passivated by Tween-20. To enable tethering to biotin-BSA, 30 μL 0.2 mg/mL neutravidin dissolved in H50 buffer (20 mM HEPES (pH 7.5) and 50 mM KCl). Non-specific sample binding to the slide was checked in the absence of neutravidin. Ribosomal complexes were imaged in polyamine buffer containing 0.8 mg/mL glucose oxidase, 0.625% glucose, 0.4 μg/mL catalase and 1.5 mM 6-hydroxy-2,5,7,8-tetramethylchromane-2-carboxylic (Trolox). A total of 2 μM of EF-G, RF3 or LepA were added to imaging buffer together with 250 μM GDPCP or GDPNP as indicated. smFRET was excited by 532 nm laser and measured by prism-type inverted TIRF (Total Internal Reflection Fluorescence) microscope with a water-immersion objective (Olympus, UplanApo, 60×, NA 1.2). A DV2 (Photometrics) dual-view imaging system equipped with a 630 nm dichroic mirror was used to split the fluorescence into Cy3 and Cy5 channels. TIRF microscopy signal was acquired by “Single” software (http://ha.med.jhmi.edu/resources/, accessed on 12 February 2013) and detected with an EMCCD camera (iXon, Andor Technology, Belfast, UK) with 100 ms integration time and with 300 EM gain.

IDL software (ITT) was used to extract fluorescence intensities of Cy3 donor (*I_D_*) and Cy5 acceptor (*I_A_*), from which apparent FRET efficiency (*E_FRET_*, hence referred as FRET) was calculated:

(1) $$E_{FRET}=\frac{I_{A}}{I_{A}+I_{D}}.$$

Traces with single-step photobleachings for both Cy5 and Cy3 were selected using MATLAB. FRET distribution histograms compiled from hundreds of smFRET traces were smoothed with a 5-point window using MATLAB and fit to two Gaussians corresponding to 0.37 and 0.56 FRET states for S6/L9 FRET Assay.

**EF-G quenching assay**. EF-G-591Cys was labeled with fluorescein maleimide and used in quenching assay as previously described [37]. Ribosomal complexes were titrated from 0 to 1.6 μM in reactions that contained 10 nM EF-G-591-fluorescein, 0.5 mM GDPNP, polyamine buffer and 0.01% Nikkol. EF-G and ribosomes were incubated for 20 min at 37 °C in a total volume of 30 μL. Fluorescence spectra (505–600 nm) were acquired using a Fluoromax 4 (Horiba, Kyoto, Japan) fluorimeter at 22 °C with excitation at 485 nm, in a 10 μL cuvette (Starna Cells, Atascadero, CA, USA). Fluorescent signal (*I*) was normalized to that of EF-G in the absence of the ribosome (*I*_0_); the K_D_ for EF-G binding was determined by fitting the data to the following equation [54]:

*I*/*I*_0_ = 1 − A× [K_D_ + P_0_ + [*Rs*] − SQRT((K_D_ + P_0_ + [*Rs*])^2^ − 4P_0_× [*Rs*])]/2 × P_0_, (2)

where P_0_ is EF-G concertation (constant), [*Rs*]—ribosome concentration (variable), A and K_D_ are amplitude and dissociation constant (parameters).

## Figures and Tables

**Table 1:** Effects of P-site tRNA deacylation and antibiotics on dissociation constants (K_D_) of EF-G binding.

P Site	Antibiotic	K_D,_ nM	*p* Value
Vacant	-	115 ± 5	
tRNA^fMet^	-	16 ± 2	
N-Ac-Met-tRNA^fMet^	-	66 ± 2	8 × 10^−7^
tRNA^Phe^	-	14 ± 2	
	Blasticidin S	37 ± 4	7 × 10^−5^
	Sparsomycin	24 ± 3	1 × 10^−3^
	Viomycin	≤5	4 × 10^−3^
N-Ac-Phe-tRNA^Phe^	-	31 ± 4	1 × 10^−3^

Dissociation constants (K_D_) of EF-G binding to ribosomes assembled with P-site peptidyl-tRNA, or antibiotics were different from those measured in ribosomes containing deacylated tRNA in the P site as p-values determined by the two-tailed Student t-test were below α (0.05). p-values were calculated relative to K_D_ values measured in ribosomes bound with either P-site tRNA^fMet^ (blue) or tRNA^Phe^ (red).

## Data Availability

All data are available upon request.
